# A new role for Zinc limitation in bacterial pathogenicity: modulation of α-hemolysin from uropathogenic *Escherichia coli*

**DOI:** 10.1038/s41598-018-24964-1

**Published:** 2018-04-25

**Authors:** Elsa Velasco, Suning Wang, Marianna Sanet, Jorge Fernández-Vázquez, Daniel Jové, Estibaliz Glaría, Annabel F. Valledor, Thomas V. O’Halloran, Carlos Balsalobre

**Affiliations:** 1 Department of Genetics, Microbiology and Statistics, School of Biology, Universitat de Barcelona, Avda. Diagonal 643, Barcelona, 08028 Spain; 2 Chemistry of Life Process Institute, and Department of Chemistry, Northwestern University, Evanston, Illinois 60208-3113 United States of America; 3 Nuclear Receptor Group, Department of Cell Biology, Physiology and Immunology, School of Biology, Universitat de Barcelona, Avda. Diagonal 643, Barcelona, 08028 Spain

## Abstract

Metal limitation is a common situation during infection and can have profound effects on the pathogen’s success. In this report, we examine the role of zinc limitation in the expression of a virulence factor in uropathogenic *Escherichia coli*. The pyelonephritis isolate J96 carries two *hlyCABD* operons that encode the RTX toxin α-hemolysin. While the coding regions of both operons are largely conserved, the upstream sequences, including the promoters, are unrelated. We show here that the two *hlyCABD* operons are differently regulated. The *hly*_*II*_ operon is efficiently silenced in the presence of zinc and highly expressed when zinc is limited. In contrast, the *hly*_*I*_ operon does not respond to zinc limitation. Genetic studies reveal that zinc-responsive regulation of the *hly*_*II*_ operon is controlled by the Zur metalloregulatory protein. A Zur binding site was identified in the promoter sequence of the *hly*_*II*_ operon, and we observe direct binding of Zur to this promoter region. Moreover, we find that Zur regulation of the *hly*_*II*_ operon modulates the ability of *E*. *coli* J96 to induce a cytotoxic response in host cell lines in culture. Our report constitutes the first description of the involvement of the zinc-sensing protein Zur in directly modulating the expression of a virulence factor in bacteria.

## Introduction

During infection, pathogens often encounter metal limitation. Alterations in the concentrations of certain metal ions have a great impact in cell physiology and gene expression. For instance, changes in the concentration of iron regulate the expression of virulence factors through metalloregulatory proteins such as Fur, the ferric uptake regulator, and the closely related PerR, the peroxide-stress regulator^1,2^. Recent studies revealed that another metal ion, zinc, also has an effect on bacterial physiology during infection^3,4^. Zinc is an essential element as a catalytic or structural cofactor of key enzymes and proteins involved in many processes such as DNA replication and protein synthesis and turnover^5,6^. Conversely, zinc can be toxic to bacteria when present in excess and so intracellular levels must be tightly controlled^5,7^. Zinc release from host tissue has been proposed to be an important innate defense mechanism^7^. In the case of *S*. *pneumoniae* infection in mice, elevated total zinc concentrations have been reported in host tissue and serum samples. The authors propose that the ability of zinc excess to inhibit pathogen growth arises from zinc competition with Mn uptake^7^. Zinc concentration in host fluids can rise in response to bacterial infection and inflammation by its release from damaged or apoptotic cells, and from sequestering proteins such as metallothionein^7^.

Studies in both *E*. *coli* and *B*. *subtilis* have shown that, in response to variations in zinc concentration, cells regulate gene expression using the metalloregulatory protein Zur (zinc uptake regulator) as a sensor. Zur forms a dimeric complex with zinc acquiring the ability to bind DNA and to repress gene expression^8^. Characterized Zur regulons mainly consist of genes coding for proteins involved in zinc homeostasis and paralogs of ribosomal proteins which, under zinc limitation conditions, replace ribosomal proteins that contain zinc^9^. Although most known Zur-regulated genes are involved in zinc uptake and adaptation to low concentrations of the metal^8–10^, a number of questions about how Zur impacts overall cell physiology and pathogenesis remain unanswered.

Uropathogenic *Escherichia coli* (UPEC) is the main causal agent of community-acquired urinary tract infections. In order to colonize its host, it expresses a battery of virulence factors including adhesins, siderophores, flagella, and toxins^11^. One of these factors is the pore-forming toxin α-hemolysin, whose expression induces tissue damage and more severe infections^12^. Approximately 40–50% of UPEC isolates are able to produce α-hemolysin^13^. Remarkably, a recent study in mice linked zinc concentrations to the pathogenic effect of α-hemolysin during gastrointestinal disorders, showing that treatment with zinc protects against α-hemolysin-induced intestinal barrier disfunction^14^.

α-hemolysin is encoded in the polycistronic operon *hlyCABD*, which contains four open reading frames (ORFs)^15^. The *hlyC* gene codes for an acyltransferase required for the activation of pro-α-hemolysin. Pro-α-hemolysin is encoded by *hlyA*, while the *hlyB* and *hlyD* genes encode two components of the secretory system that transports α-hemolysin out of the cells^16,17^. The *hlyCABD operon* in *E*. *coli* is tightly regulated and strongly affected by several environmental and physiological signals. A number of regulators, including RfaH, CpxRA and H-NS, modulate α-hemolysin expression^18–21^. In hemolytic UPEC isolates from humans, the *hlyCABD* operon is present in the chromosome within pathogenicity islands, together with genes coding for other virulence factors^22,23^. Although most hemolytic UPEC strains carry a single copy of the operon, the presence of two functional copies of the *hlyCABD* operon has been detected in two pyelonephritis isolates, J96 and 536^23^.

In this article, we show that the two hemolytic operons present in the chromosome of *E*. *coli* J96 are differentially regulated. The *hly*_*I*_ operon does not respond to zinc limiting conditions whereas the *hly*_*II*_ operon is silenced in the presence of zinc in a Zur-dependent manner. A highly conserved Zur binding site was found in the promoter sequence of the *hly*_*II*_ operon. Direct binding of Zur to this promoter was confirmed by electrophoretic mobility shift assay (EMSA). Although a role of Zur-mediated regulation in bacterial pathogenicity has previously been suggested^6,24^, our research represents the first description of the involvement of zinc and the zinc-sensing transcription factor Zur in regulating the expression of a virulence factor in bacteria directly.

## Results

### The two hemolytic operons of J96 are differentially expressed

**Figure 1:**
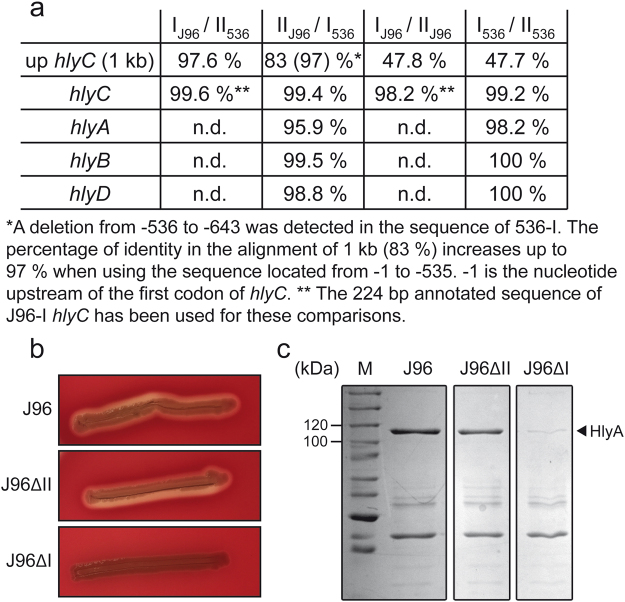
Differential expression of the two *hlyCABD* operons present in the J96 strain. (**a**) Percentage of identity at the level of the nucleotide sequence between the operon *hly*_*I*_ and *hly*_*II*_ of J96 and 536. n.d.: not determined. (**b**) Hemolytic phenotype of J96, JFV16 (J96ΔII) and JFV21 (J96ΔI) strains on Columbia Blood agar plates. (**c**) Coomassie blue stained SDS-PAGE (10%) of secreted protein extracts from cultures grown in LB_0_ at 37 °C up to late-log phase (OD_600 nm_ of 1.0) of the indicated strains. Lane M: molecular mass markers (size in kDa indicated along the left side). The band corresponding to α-hemolysin (HlyA) is indicated with an arrowhead. Full-length gel image is shown in Fig. S6.

Two UPEC isolates, J96 and 536, extensively used as model organisms in pathogenicity studies, have two functional *hlyCABD* operons (designated I and II) coding for α-hemolysin (HlyA)^23^. The full sequences of the two operons from 536 and the *hlyCABD*_*II*_ operon (*hly*_*II*_) from J96 are known; however, only a fragment of the *hlyCABD*_*I*_ operon (*hly*_*I*_) from J96, carrying part of the *hlyC* gene (224 bp) and its upstream sequence, is available. While the ORFs of the sequenced operons are almost identical, the *hlyC* upstream sequences carrying the promoter and the regulatory motifs diverge extensively between operons (Fig. 1a). Consistent with a previous report^23^, pairwise alignments showed two different types of sequence upstream of *hlyC* and indicated that the *hly*_*II-*J96_ operon is identical to the *hly*_*I-*536_ operon and that *hly*_*I*-J96_ is identical to *hly*_*II*-536_. The *hly*_*II-*J96_ operon was the first *hlyCABD* operon cloned from human isolates and used to substantiate the contribution of HlyA to UPEC virulence^25,26^.

J96 derivative mutant strains carrying partial deletions of either *hly*_*I*_ or *hly*_*II*_ were obtained by gene replacement. The hemolytic phenotype of the mutant derivatives was monitored on Columbia Blood Agar (CBA) plates (Fig. 1b). The mutant J96ΔII (Δ*hly*_*II*_), carrying only the *hly*_*I*_ operon, displays a phenotype identical to the J96 strain. However, the J96ΔI (Δ*hly*_*I*_), carrying only the *hly*_*II*_ operon, exhibits a very thin hemolytic halo, suggesting that very little HlyA is produced from *hly*_*II*_ operon under optimal conditions for HlyA detection^27^. The levels of secreted HlyA by J96 and its derivatives J96ΔI and J96ΔII were determined in bacterial cultures grown in LB_0_ (with no NaCl added) at 37 °C up to late-logarithmic phase. SDS-PAGE separation of secreted protein extracts from J96 revealed a single band at 110 kDa, corresponding to HlyA. Consistent with the hemolytic phenotype, the amount of HlyA secreted by the J96ΔII strain (*hly*_*I*_ operon) was very similar to the amount detected in the extract from the wild-type strain, whereas the amount of HlyA secreted by the J96ΔI strain (*hly*_*II*_ operon) was much lower (Fig. 1c). These results suggest that in the assessed experimental conditions, the *hly*_*I*_ operon is the main contributor to the HlyA production and the *hly*_*II*_ operon is somehow silenced. The extracts analyzed in Fig. 1c were obtained from cultures grown in LB_0,_ however, the described differential expression of the two *hly* operons of J96 does not depend on the external osmolarity. Secreted protein extracts from cultures grown in LB (5 g l^−1^ of NaCl) were also analyzed. Although expression of HlyA was lower in all the strains, *hly*_*I*_ expressed most of the HlyA, while expression of HlyA from the *hly*_*II*_ operon was barely detected (Fig. S1).

### A mutation in *zur*, encoding the zinc uptake regulator, causes derepression of the *hly*_*II*_ operon

**Figure 2:**
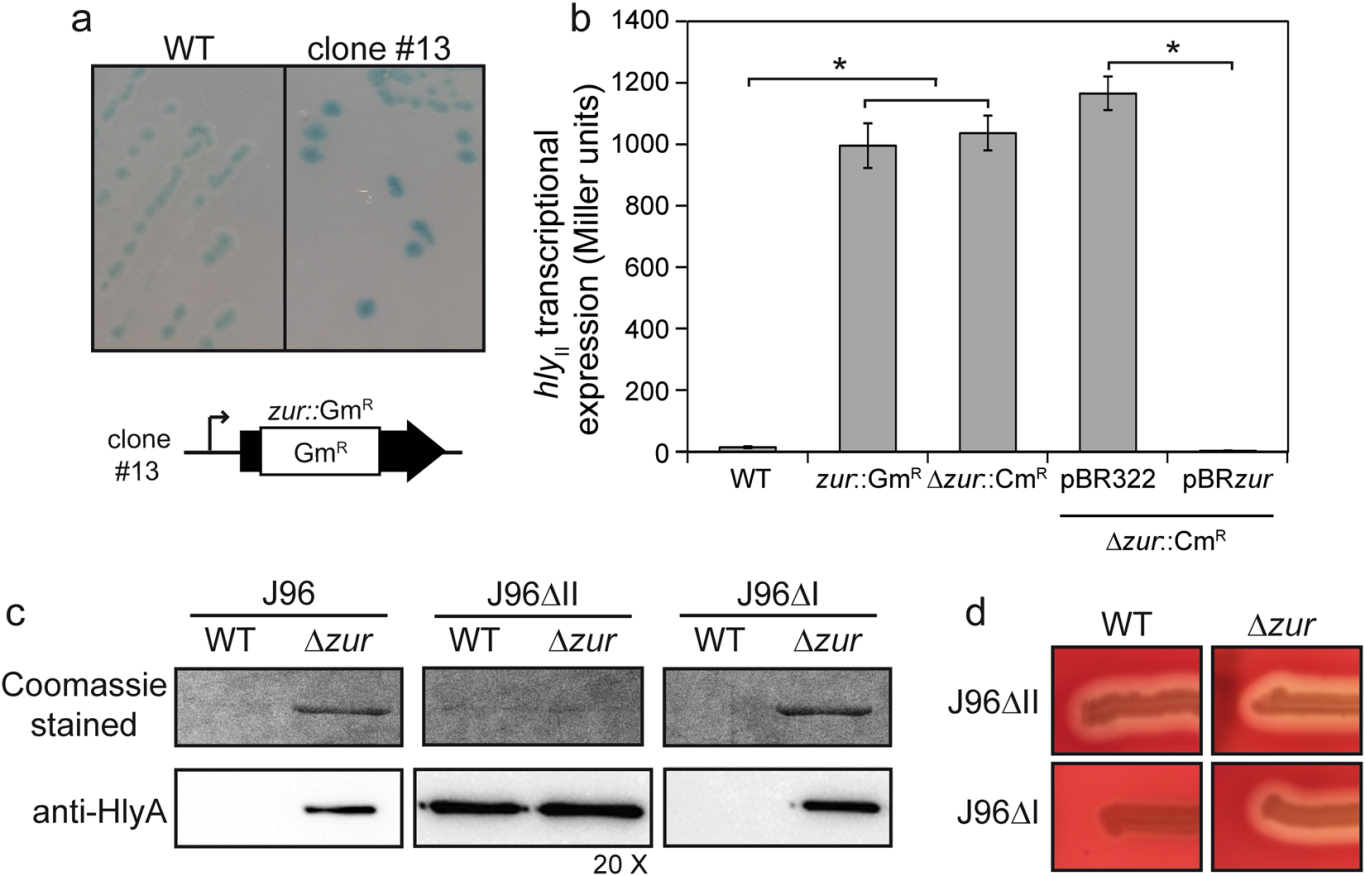
Expression of the *hly*_*II*_ operon of J96 is derepressed by a mutation in the *zur* gene. (**a**) Lac-phenotype on LB Xgal agar plates of the JFV3 (J96 *hly*_*II*_::*lacZ*) strain and its Gm^R^ derivative (clone #13) obtained by random mutagenesis. Below is represented the location of the Gm^R^ transposon insertion in clone #13. (**b**) Transcriptional expression of the *hly*_*II*_ operon from cultures of the JFV3 strain and the two derivatives *zur::*Gm^R^ (clone #13) and *zur::*Cm^R^ (EV46) grown in LB at 37 °C up to late-log phase (OD_600 nm_ of 1.0). β-Galactosidase activity (Miller units) was determined in three independent cultures. Mean values with standard deviations are plotted. *P < 0.05, ANOVA with Tukey’s multiple comparisons test. (**c**) Electrophoretic analysis of secreted protein extracts from cultures grown in LB at 37 °C up to late-log phase of J96, JFV16 (J96ΔII) and JFV21 (J96ΔI) strains and their otherwise isogenic *zur::*Cm^R^ mutants (EV27, EV34 and EV38, respectively). Upper panels are Coomassie blue stained 10% SDS-PAGE and lower panels immunodetection using monoclonal anti-HlyA antibody. Samples for immunodetection from J96ΔII were concentrated 20x with respect to rest of samples for better visualization. Full-length gel and blot images are shown in Fig. S7. (**d**) Hemolytic phenotype of JFV16 (J96ΔII) and JFV21 (J96ΔI) strains and its *zur* mutant derivatives on Columbia Blood agar plates.

To identify putative regulators of the *hly*_*II*_ operon responsible for its low expression under the assessed conditions, random mutagenesis using a gentamycin resistance mariner transposon was performed on strain JFV3, a J96 derivative that carries a chromosomal *hly*_*II*_*::lacZ* reporter fusion. Mutants were selected according to their Lac phenotype on LB agar plates with X-gal, to detect *hly*_*II*_*::lacZ* expression. The clone #13 was selected for its deep blue colour compared to the parental strain, which indicates increased expression of *hly*_*II*_*::lacZ* (Fig. 2a). Quantitative expression studies showed that the selected Gm^R^ mutant causes a drastic derepression (47-fold) of the *hly*_*II*_ operon transcriptional expression (Fig. 2b). Using primers for the mariner transposon, the nucleotide sequence adjacent to the insertion was determined in clone #13. The transposon was inserted within the *zur* gene, in codon 142 of 172.

The *zur* gene encodes the Zur protein (zinc uptake regulator), a transcriptional repressor that binds to DNA in the presence of zinc and thereby represses specific genes^28^. To corroborate the association of Zur with the observed derepression, a new *zur* mutant was generated in J96 by gene replacement. The sequence of the *zur* gene between positions 43 and 381 bp (position 1 corresponds to the first nucleotide of the first codon) was deleted, and a Cm^R^ cassette was inserted. The *zur::*Cm^R^ allele causes the same effect on *hly*_*II*_ transcriptional expression as the Gm^R^ allele obtained by random mutagenesis both in late-logarithmic phase (Fig. 2b) and in mid-logarithmic phase (Fig. S2) of bacterial growth. The effect of the *zur* mutation on *hly*_*II*_ expression was also determined by measuring the amount of the secreted HlyA in culture supernatants of Zur proficient and deficient derivatives of J96, J96ΔI and J96ΔII strains. Both Coomassie-staining and immunodetection using specific HlyA antibodies showed a clear increase in the amount of HlyA in the presence of the *Δzur* mutation in the two strains carrying the *hly*_*II*_ operon (J96 and J96ΔI) (Fig. 2c). By contrast, derepression of the *hly*_*I*_ operon (J96ΔII strain) was not detected in the *zur* mutant (Fig. 2c and Fig. S1). Due to the low expression level of the *hly*_*I*_ operon in LB, HlyA immunodetection in J96ΔII extracts was performed on 20-fold concentrated samples. Furthermore, the severity of the hemolytic phenotype correlates with the amount of HlyA in each strain. The strain carrying only the operon *hly*_*II*_ showed minimal hemolysis in the presence of an intact *zur* gene and a higher hemolytic phenotype when the *zur* gene was mutated. Strains carrying only the *hly*_*I*_ operon showed the same phenotype, regardless of the presence or absence of an intact *zur* gene (Fig. 2d). These results clearly indicate that Zur specifically regulates the *hly*_*II*_ operon. Zur-mediated regulation of *hly*_*II*_ transcription was confirmed by trans-complementation experiments (Fig. 2b). The strong derepression of the *hly*_*II*_ operon observed in the *zur* mutant was fully complemented when a pBR322 derived plasmid containing the J96 *zur* gene with its own promoter was introduced in the mutant strain. Our findings on the Zur-mediated regulation of the *hly*_*II*_ operon of J96 are the first description of a regulatory factor that modulates differently the two hemolytic operons present in J96.

**Figure 3:**
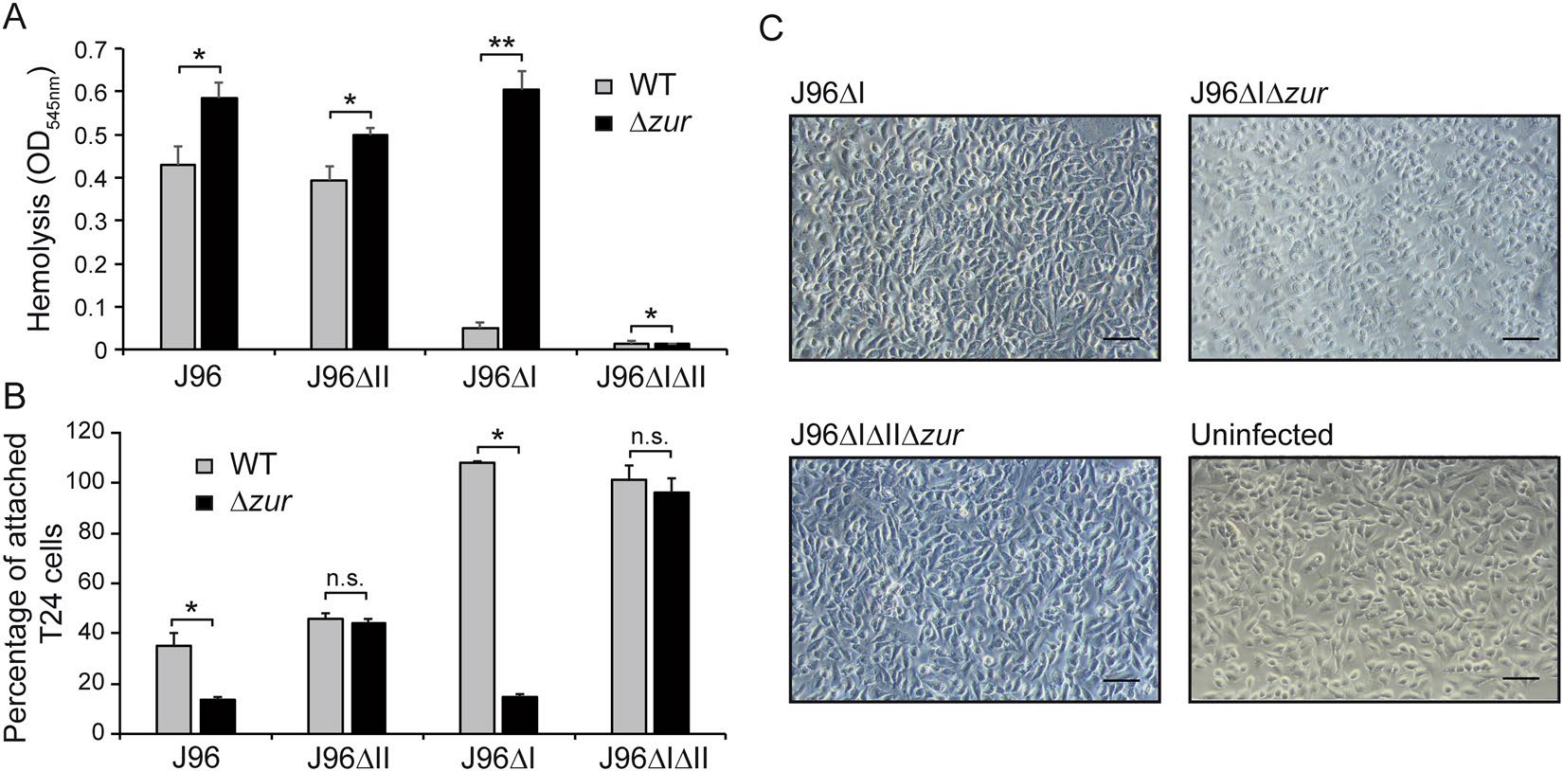
Effect of *zur* mutation in UPEC virulence-associated phenotypes. (**A**) Hemolysis after 1 hour infection of sheep blood cell suspensions with J96, JFV21 (J96ΔI), JFV16 (J96ΔII) and EV64 (J96ΔIΔII) strains and their otherwise isogenic *zur* mutants (EV27, EV34, EV38 and EV65). The release of haemoglobin was monitored by OD_545 nm_. (**B**) T24 cell monolayer detachment activity. Percentage of remaining attached T24 cells, measured as OD_590 nm_, after 3.5 h post-infection with the same strains as in (**A**) Mean values with standard deviation from three independent experiments (**A**) and three biological replicates (**B**) are plotted. *P < 0.05 **P < 0.0001 n.s.: non-significant, t test with p-values adjusted by Bonferroni’s method (**C**) Phase contrast microscopy of T24 bladder epithelial cell monolayers infected for 2 hours with the indicated strains. Scale bar, 0.1 mm.

Hemolysin has a role in cell toxicity and urothelial damage during infection^19,29^. In order to determine the impact of Zur-mediated regulation of α-hemolysin on bacterial pathogenicity, we assessed the ability of different derivatives of the J96 strain to induce hemoglobin release after infection of blood cell suspensions and cell detachment after infection of bladder epithelial cell monolayers. A slight increase in hemolytic activity was detected after infection of the Δ*zur* derivatives of J96 and J96ΔII as compared to the *zur*^+^ counterparts (Fig. 3A). Remarkably, the *zur* mutation caused a very drastic effect when blood cell suspensions were infected with derivatives of the J96ΔI strain. Hemolytic activity was more than 12-fold higher in the Δ*zur* mutant as compared to wild-type strain, consistent with the described derepression of the *hly*_*II*_ operon in strains lacking Zur. As a control, J96 derivative strains lacking both *hly*_*I*_ and *hly*_*II*_ operons showed very low hemolysis in either *zur*^+^ or Δ*zur* background. These results clearly demonstrate that the effect observed was caused by α-hemolysin.

Next, we performed infection assays of bladder epithelial cells (T24 monolayers) using the same bacterial strains (Fig. 3B). After 3.5 hours of infection with the J96ΔI strain, the monolayers were intact, showing a percentage of attached cells similar to the uninfected monolayers. In contrast, when monolayers were infected with the Δ*zur* derivative of J96ΔI less than 20% of the cells remained attached after 3.5 hours. Again, the effect can be attributed to the production of α-hemolysin since cell attachment did not decrease after infection with J96ΔIΔIIΔ*zur*. The specific effect of α-hemolysin production on cell monolayers could be detected 2 hours post-infection as cells became round-shaped when infected with strain J96ΔIΔ*zur* (Fig. 3C), an α-hemolysin-induced phenotype previously described^30^. Both wild-type and Δ*zur* derivatives of the J96 and J96ΔII strains, carrying the Zur-independent *hly*_*I*_ operon, disrupt cell adhesion at different extents (Fig. 3B). In line with the aforementioned expression studies on the *hly*_*II*_ operon, the Δ*zur* mutation triggers virulence-associated features such as hemoglobin release and disruption of epithelial cell adhesion.

### Expression of the *hly*_*II*_ operon responds to the presence of zinc

**Figure 4:**
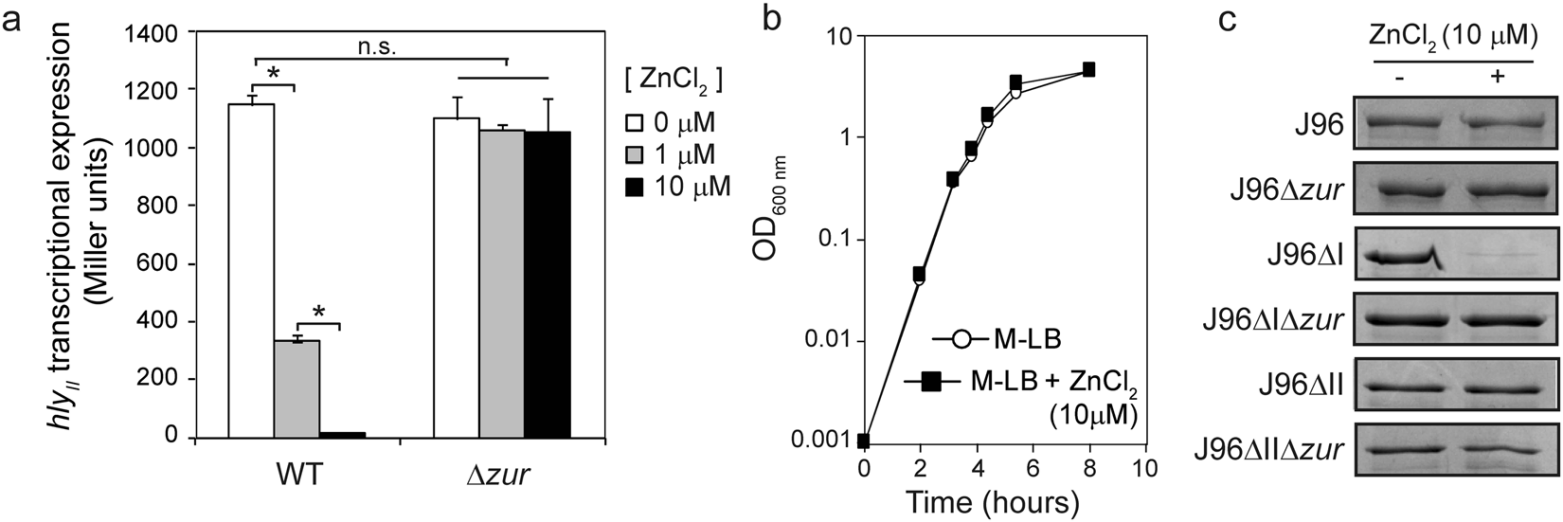
The expression of the *hly*_*II*_ operon of J96 responds to the external levels of zinc. (**a**) Transcriptional expression from the *hly*_*II*_ promoter in cultures of the strains JFV3 and its *zur::*Cm^R^ mutant derivative (EV46) grown in M-LB and M-LB replenished with either 1 or 10 μM ZnCl_2_. Culture samples were taken at late-log growth phase (OD_600 nm_ of 1.0). β-galactosidase activity (expressed in Miller units) was determined from three independent cultures; mean values with standard deviation are plotted. *P < 0.05, ANOVA with Tukey’s multiple comparisons test (**b**) Growth curves of JFV3 strain cultured in M-LB and in M-LB replenished with 10 μM ZnCl_2_. Growth was monitored by measuring OD_600 nm_ from two independent cultures; mean values are plotted.(**c**) Detection of the α-hemolysin in secreted protein extracts from cultures of J96, JFV21 (J96ΔI) and JFV16 (J96ΔII) strains and their otherwise isogenic *zur* mutants (EV27, EV34 and EV38) grown in M-LB and M-LB replenished with 10 μM ZnCl_2_. Full-length gel images are shown in Fig. S8.

In the presence of zinc, the transcriptional regulator Zur forms a complex that binds to DNA, causing transcriptional repression^8^. We hypothesized that the *hly*_*II*_ operon should be repressed by the presence of zinc in a Zur-dependent manner. To test this hypothesis, transcriptional expression of the *hly*_*II*_ operon was monitored in cultures of strain JFV3 grown in either LB depleted from zinc (M-LB) or the same medium replenished with 1 μM and 10 μM ZnCl_2_. In the *zur*^+^ strain, *hly*_*II*_ transcriptional expression is high in the absence of zinc and severely drops in medium replenished with this metal ion (Fig. 4a). Remarkably, the response of *hly*_*II*_ expression to zinc was dose-dependent. Furthermore, zinc-mediated regulation is strictly dependent on the presence of Zur, since no repression is observed in a *zur* mutant strain by the presence of zinc. The growth kinetics of the JFV3 strain was not altered by the absence or presence of different zinc concentrations (Fig. 4b). The effect of zinc on *hly*_*II*_ transcriptional expression was corroborated by monitoring the level of secreted HlyA. In cultures of the strain J96ΔI, a high amount of secreted HlyA was detected in the absence of zinc, whereas the HlyA amount was very low in cultures grown in the presence of ZnCl_2_ (10 μM) (Fig. 4c). The amount of secreted HlyA from *hly*_*I*_ operon was also monitored in both the absence and presence of zinc. Consistent with the fact that *hly*_*I*_ operon expression was not altered by the *zur* mutation, the expression of *hly*_*I*_ operon was not affected by the presence of zinc. In cultures of the J96 strain, carrying both *hly*_*I*_ and *hly*_*II*_ operons, a slight effect on HlyA production by the presence of zinc was detected.

### Zur directly represses *hly*_*II*_ expression by binding to its promoter

**Figure 5:**
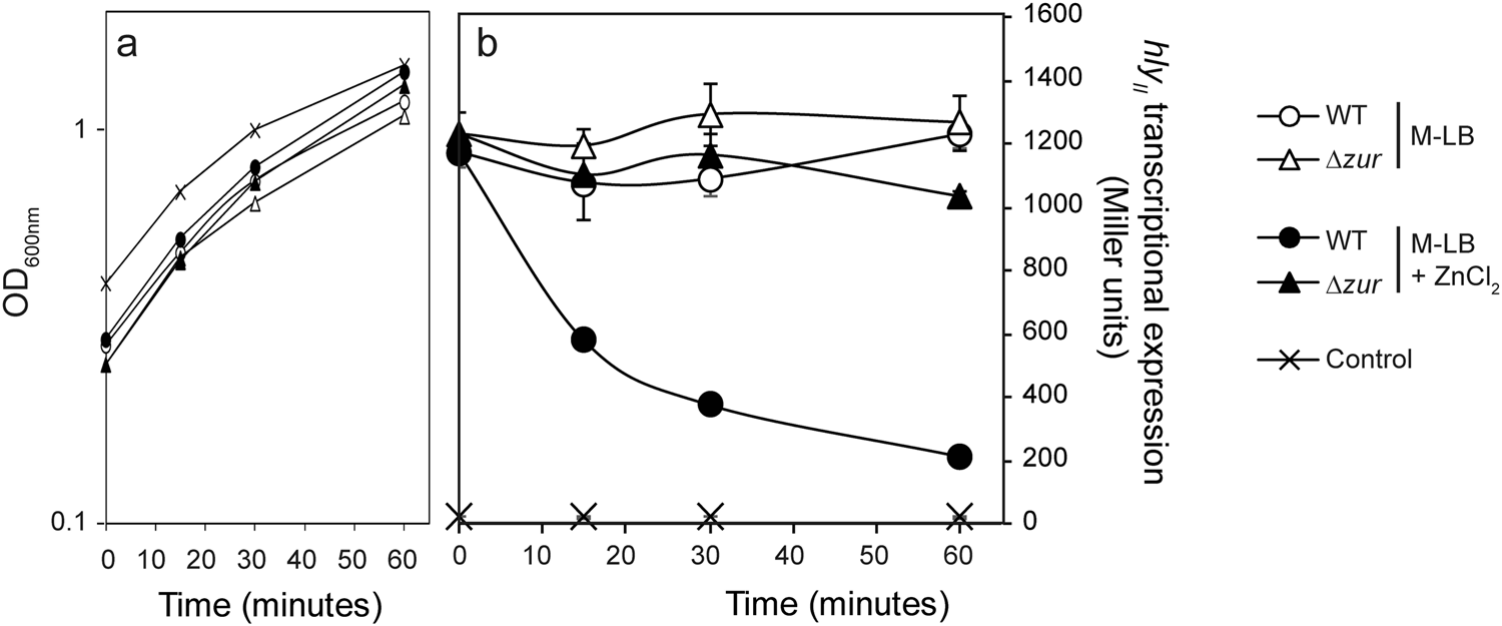
Rapid response of *hly*_*II*_ transcriptional expression to zinc addition. (**a**) Growth curves of the strain JFV3 and EV46 (*zur::*Cm^R^) in the indicated media by measuring OD_600 nm_ from two independent cultures; mean values are plotted. (**b**) Transcriptional expression of *hly*_*II*_ in cultures of JFV3 and EV46 after zinc addition. Cultures were grown in M-LB up to an OD_600 nm_ of 0.3 when media was either replenished or not with 10 μM ZnCl_2_. Culture samples were taken right before ZnCl_2_ addition (0 minutes), and after 15, 30 and 60 minutes. As a control, JFV3 was cultured in M-LB replenished with 10 μM ZnCl_2_ from the beginning. β-galactosidase activity (expressed in Miller units) was determined from three independent cultures; mean values with standard deviation are plotted.

The effect of sudden addition of ZnCl_2_ (10 μM) on *hly*_*II*_ transcriptional expression was monitored in cultures of strains JFV3 and its *∆zur* derivative grown in M-LB medium up to an OD_600 nm_ of 0.3. Bacterial growth was not substantially affected by adding ZnCl_2_ (10 μM) to the zinc-free medium cultures (Fig. 5a). The ZnCl_2_ exposure caused a rapid decrease of the *hly*_*II*_ transcriptional expression in the Zur proficient strain, as compared to cultures where no ZnCl_2_ was added (Fig. 5b). After 15 minutes, a drop in *hly*_*II*_ expression was already detected, falling to 53% of the levels measured in cultures grown with no ZnCl_2_ addition. Expression progressively declined to 20% after one hour exposition to ZnCl_2_. In agreement with all previous results, exposure to ZnCl_2_ during one hour did not significantly alter the expression of the *hly*_*II*_ operon in a *zur* mutant strain. As a control, the level of *hly*_*II*_ operon expression was very low in cultures grown in M-LB replenished with ZnCl_2_ from the start.

**Figure 6:**
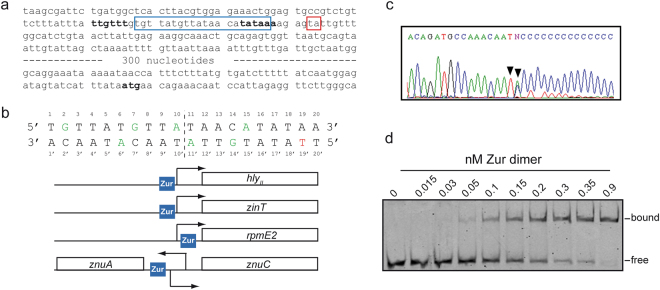
Zur protein binds to the *hly*_*II*_ promoter. (**a**) Sequence of the *hly*_*II*_ operon upstream of *hlyC*. In bold are indicated the first codon (ATG) of *hlyC* and the putative −10 and −35 boxes of the promoter. Within the blue box is indicated the putative binding site of Zur. The red box indicates the transcriptional start found by 5′RACE assays. (**b**) Sequence of the putative Zur binding site found in the *hly*_*II*_ operon promoter. Bases recognized by Zur dimers are colored: green for conserved bases and red for the only base that is not conserved. A dashed line represents the symmetry axis. Below are depicted the positions of Zur binding sites in promoters of *znuABC*, *zinT*, *rpmE2*^9^ and *hly*_II_. Zur boxes were drawn to scale in respect of transcription start site. (**c**) Sequence of the 5′RACE assay using J96 total RNA samples grown in LB at 37 °C up to late log phase. The two bases identified as a transcriptional start are indicated with arrowheads. The N residue corresponds to a mix of A and C bases. (**d**) Electrophoretic mobility shift assay. Titration of a Cy5 labeled 50-bp *hly*_*II*_ promoter fragment carrying the putative Zur binding site was carried out in the presence of excess salmon sperm DNA which serves as a non-specific competitor. Samples contains 60 pM DNA, plus 0, 15, 30, 50, 100, 150, 200, 300, 350, 900 pM Zur (calculated as dimer), respectively. Samples were resolved on a 10% polyacrylamide gel. Both gel and electrophoresis buffers contain 50 µM ZnSO_4_. Full-length gel image is shown in Fig. S9.

The fast response to ZnCl_2_ of *hly*_*II*_ transcriptional expression suggested that Zur directly represses the *hly*_*II*_ promoter. Zur regulates transcription by cooperative binding of two dimers to a well-characterized DNA sequence located in the vicinity of the promoter of Zur-regulated genes^9,31^. Using the consensus sequence reported^31^ for the *E*. *coli* Zur box (RNNNYRNNRYNNYRNNNY), the J96 *hly*_*II*_ promoter sequence was scrutinized, revealing a putative Zur binding site (Fig. 6a and b). The bases of the Zur box involved in establishing hydrogen-bonds with Zur have been identified^31^ and are indicated in Fig. 6b. The motif found in the J96 *hly*_*II*_ promoter sequence was highly conserved except for the base G19′ (see Fig. S3). It must be noted that G19′ is one of the least determinant bases for Zur binding^31^.

A transcriptional start for the *hly*_*II*_ operon was previously proposed based on primer extension analyses on RNA samples from K12 strains carrying the *hly*_*II*_ operon in a multicopy plasmid^32^. In our study, 5′-RACE with RNA samples from J96 was performed and a *hly*_*II*_ operon transcription start was defined 9 nucleotides upstream of the previously described +1 position. This transcriptional start site is properly positioned to a putative promoter identified with the Neural Network Promoter Prediction software (Fig. 6a and c). Consequently, the putative Zur binding site is located at positions −25 to −6. Zur binding would overlap the −10 sequence, blocking the binding of RNA polymerase and repressing *hly*_*II*_ transcription. The relative position of the putative Zur binding site found in the *hly*_*II*_ operon and other genes of the *E*. *coli* Zur regulon was compared after searching the Zur consensus sequence described^31^ within the upstream regions of Zur-regulated genes^9^. The relative location of the putative Zur binding site in the *hly*_*II*_ operon turned out to resemble the position of other members of the Zur regulon (Fig. 6b). For instance, the location of the Zur box in *znuC* and *zinT* is −22 to −3 and −23 to −4, respectively.

The ability of Zur to bind to the *hly*_*II*_ promoter sequences *in vitro* was assessed using EMSA. The Zur protein used in this assay is derived from the *E*. *coli* strain MG1655, which shares 97.7% identity and 100% similarity with the Zur protein from J96. The results clearly demonstrate that Zur binds the *hly*_*II*_ promoter *in vitro* (Fig. 6d). Remarkably, *E*. *coli* Zur binds to the *hly*_*II*_ operon even more tightly than it does to the *E*. *coli* MG1655 Zur promoter region for the *L31p* promoter (0.025 ± 0.01 × 10^−18^ M^2^), which corresponds to the highest affinity of any of the operator sites evaluated^31^. While the binding is too tight to calculate a reliable dissociation constant under these experimental conditions, the Zur concentration at half maximal binding (100 nM Zur dimer) allow us to estimate an upper limit for this dissociation constant, i.e. <0.02 × 10^−18^ M^2^. Together with the *in vivo* studies on gene expression, these data reveal that Zur likely represses transcription of the *hly*_*II*_ operon directly and confirm that the *hly*_*II*_ operon is part of the Zur regulon in the UPEC strain J96. Interestingly, the putative Zur binding site was also found in the *hly*_*I*_ operon from the UPEC strain 536 that is equivalent to *hly*_*II*_ from J96. In 536 two mismatches from the consensus sequence were found: the mismatch already found in J96 *hly*_*II*_ and a second base which does not directly interact with Zur (in position 13, a G instead of A or T is found). The upstream intergenic sequence of the *hly*_*I*_ operon of J96 was also analyzed for a Zur binding site and, as expected, none was found. No Zur binding sites were found in upstream intergenic regions of *hly* operons from other UPEC isolates such as CFT073, UTI89 and F11. Pairwise alignments showed that these operons and their promoters are J96 *hly*_*I*_-like. Accordingly, the Δ*zur* mutation in CFT073 does not cause an increase of HlyA production and hemolysis after infection of blood cells (Fig. S4).

## Discussion

The presence of two *hlyCABD* operons in the UPEC isolates J96 and 536, with more than 7 Kb DNA sequence sharing 99% identity, is intriguing considering that the presence of large homologous DNA sequences in a chromosome may cause genome instability and result in detrimental effects on bacterial fitness. Interestingly, the sequences upstream of the *hlyC* gene from both operons differ greatly, indicating that their regulatory sequences are unrelated and suggesting the existence of different mechanisms of regulation. Indeed, in this report we demonstrate that the *hly*_*II*_ operon is silenced by the Zur regulator allowing expression only under zinc limiting conditions. In contrast, the operon *hly*_*I*_ does not respond either to zinc or this regulator (Zur). Interestingly, most *hlyCABD* operons from human isolates described in databases showed homology to the Zur-insensitive operon *hly*_*I*_, whereas the *hly*_*II*_ operon was only detected in two pyelonephritis isolates, 536 and J96, and in the environmental isolate MRE600. Recent genomic and phylogenetic studies revealed that MRE600 is an *E*. *coli* strain with distinctive genomic properties and more similar to *Shigella* than to other *E*. *coli* strains^33^.

One may hypothesize that expressing hemolysin under a wide variety of environmental conditions, including zinc limitation, may be beneficial to ensure success during the process of host colonization. In this scenario, the versatility provided by having two operons that respond to different conditions may constitute an adaptive advantage that outweighs the detrimental effect in fitness caused by genome instability due to the coexistence of large and highly homologous DNA sequences.

Previous studies suggest that zinc may play a relevant role during host colonization since zinc uptake systems are important for fitness in different pathogens, including UPEC^6,34^. However, the exact role of the Zur protein in the control of virulence factors has not yet been elucidated^35^. In *S*. *aureus*, Zur does not have an apparent role in pathogenicity, as concluded from assays using an *in vivo* infection model^36^. In *M*. *tuberculosis* and *S*. *suis*, Zur regulates genes coding for proteins belonging to the early secretory antigen target and metalloproteases, respectively, although the role of those proteins in virulence is unclear^37,38^. In *A*. *baumannii*, the *zur* mutant exhibits a defect in dissemination in a pneumonia mouse model, and RNA-seq assays showed that several genes encoding secreted proteins and putative virulence factors were upregulated in the mutant strain^24^. Our study is the first description of a direct Zur-mediated regulation of the expression of a virulence factor, UPEC’s α-hemolysin. More precisely, Zur represses the transcriptional expression of the *hly*_*II*_ operon of J96, which consequently is only expressed under zinc limiting conditions. This finding raises thought-provoking questions. Is α-hemolysin expression a mechanism used by the bacterium to release or mobilize zinc from cells? If so, this may facilitate growth of bacterial pathogens in spite of host-induced zinc limitation. In this scenario, *hly*_*II*_ expression would be acting as a bacterial factor involved in zinc homeostasis. Is the response of the J96 *hly*_*II*_ operon to zinc, and therefore the acquisition of the Zur box, required for proper sensing of specific environments? If so, the presence of *hly*_*II*_ may facilitate efficient colonization of sites within the host.

As exemplified by iron, metal limitation is a common situation during host-pathogen interactions^1^. Zinc concentration in most human tissues is extremely low^39^. Within the cytosol of human cells available zinc is particularly scarce, due to its sequestration by metallothioneins^40^. Furthermore, during infection phagocytes and epithelial cells express other zinc-chelating proteins, such as calprotectin^41–43^. In the case of UPEC, which invades the urinary tract, it inevitably faces zinc-limiting conditions since zinc concentration in human urine was reported to range between 0.17 and 0.5 ppm (2.6 to 7.6 µM) in healthy subjects, being one of the human corporal fluids with lowest zinc concentration reported^39,44,45^. Furthermore, UPEC probably encounters zinc starvation during intracellular growth, where bioavailable zinc is limited due to sequestration by host proteins like metallothioneins.

On the other hand, zinc concentration in the intestine, the reservoir of UPEC, is considerably high, due to the predominant fecal excretion of this metal^46^. One may speculate that during intestinal transit *hly*_*II*_ expression would be repressed by Zur, and therefore very little HlyA would be produced from this operon. Interestingly, during acute gut inflammation, calprotectin released by neutrophils sequesters zinc and, during this zinc-limiting conditions, *Salmonella enterica* Typhimurium grows better than gut commensal bacteria due to increased expression of the Zur-regulated ZnuABC transporter^3^. Moreover, anomalously high expression of *hlyA* by the UPEC isolate 536 in the intestine has been detected during active ulcerative colitis, which produces acute inflammation^47^. Therefore, it is reasonable to hypothesize that intestinal zinc limitation due to inflammation could promote the production of HlyA by UPEC strains residing in the gut and carrying hemolytic operons responsive to zinc, thus producing further inflammatory lesions in ulcerative colitis. A recent report described zinc treatment to be efficient against α-hemolysin-induced intestinal leakage in mice colonized by the pyelonephritis isolate 536^14^. It is known that zinc is an important factor for intestinal barrier integrity^48^. Although the authors showed that zinc has a protective effect on α-hemolysin-mediated damage *per se*, our data indicate that zinc may have an additional protective effect by promoting downregulation of α-hemolysin expression from the 536 strain, which contains a homolog of the Zur-regulated operon here reported. Our study describes the direct involvement of Zur in the control of virulence expression and suggests that zinc limitation may play a pivotal signaling role during the infectious process.

## Methods

### Bacterial strains, plasmids and growth conditions

Bacterial strains used in this work, shown in Table 1, are all derived from the pathogenic isolates J96 and CFT073. All strains were grown routinely in LB (Lennox broth, tryptone 10 g l^−1^, yeast extract 5 g l^−1^ and NaCl 5 g l^−1^) at 37 °C with aeration. LB without NaCl and with 10 g l^−1^ of NaCl was used and denoted LB_0_ and LB_10_, respectively. In most experiments, bacterial cultures were grown up to an OD_600 nm_ of 1.0, denoted late-log phase of growth. The zinc-free medium (M-LB) was prepared as indicated^49^. Briefly, LB medium was treated with 50 mg l^−1^ of chelating resin Chelex 100 (Bio-Rad) one hour at room temperature while stirring. After removing the resin by filtration (0.2 μm), trace elements were replenished by adding 1 μM FeCl_3_, 1 μM CaCl_2_, 20 μM MgCl_2_ and 1 ml l^−1^ SL-7 trace element solution (without zinc). Finally, 20 μM TPEN (N,N,N′,N′-Tetrakis(2-pyridylmethyl)ethylenediamine, Sigma-Aldrich) was added to further chelate zinc. Replenishment solutions were prepared out of concentrated stock solutions and sterilized by filtration using 0.2 μm filters when necessary. SL-7 trace element solution without zinc has the following composition: 10 ml 25% HCl, 1.5 g FeCl_2_·4H_2_O, 190 mg CoCl_2_·6H_2_O, 100 mg MnCl_2_·4H_2_O, 63 mg H_3_BO_3_, 36 mg Na_2_MoO_4_·2H_2_O, 24 mg NiCl_2_·6H_2_O, 17 mg CuCl_2_·2H_2_O and deionized water to a final volume of 1 L. In order to minimize contamination by zinc, all cultures with M-LB were performed in 5 ml culture medium using 50 ml conic centrifuge plastic tubes. Any other used material was also made of plastic and handled so as to avoid metal contamination. To monitor hemolytic phenotype Columbia Blood Agar plates (Scharlau) were used.

**Table 1:** Strains used in this work.

Strain	Genotype
J96	Pathogenic isolate
JFV23	J96 Δ*lac*
EV27	J96 Δ*zur::*Cm^R^
JFV16	J96 Δ*hly*_II_
EV34	JFV16 Δ*zur::*Cm^R^
JFV3	JFV23 *hlyA::lacZ* Km^R^
Clone #13	JFV3 *zur::*Gm^R^
EV46	JFV3 Δ*zur::*Cm^R^
JFV21	J96 Δ*hly*_I_
EV38	JFV21 Δ*zur::*Cm^R^
EV64	J96 Δ*hly*_II_ Δ*hly*_I_:: Cm^R^
EV65	J96 Δ*hly*_II_ Δ*hly*_I_:: Cm^R^ Δ*zur::*Km^R^
CFT073	Pathogenic isolate
DJ1	CFT073 Δ*zur::*Cm^R^

When needed, antibiotics were added to culture media at the indicated concentrations: kanamycin (50 μg ml^−1^), ampicillin (50 μg ml^−1^), chloramphenicol (25 μg ml^−1^) and gentamycin (10 μg ml^−1^). X-gal (5-bromo-4-chloro-3-indoyl-β-D-galactopyranoside) was added to the medium at 40 μg ml^−1^.

### Genetic techniques

The primers used in this work are listed in Table S1 (supplementary material). The strain J96Δlac is a Δ*lacZ* derivative of J96 strain obtained as previously described^50^. The strain JFV16 (J96ΔII), carrying a *hlyA* deletion from codon 20 to 434 was constructed using the primers HlyA-P1 and HlyA-P2 and the method described by Datsenko and Wanner^51^. Strain JFV21 (J96ΔI), carrying a deletion from upstream of *hlyC* to the codon 422 of *hlyA* was constructed using the same methodology and the primers Hly1-P1 and Hly1-P2-2. Strain EV64 (J96ΔIΔII) was obtained using the same methodology and the primers Hly1-P1 and Hly1-P2-2 on the JFV16 strain. Similarly, *zur* mutants were generated using primers ZurP1 and ZurP2.2 in J96 and ZurP1.2 and ZurP2.1 in CFT073. The strain JFV3 carries a promoter less *lacZ* gene linked to a kanamycin resistance cassette inserted in the *hlyA* gene of the *hly*_*II*_ operon. The *lacZ*-Km^R^ cassette from plasmid pKG137 was inserted into the *hlyA* gene of JFV16 following the method described previously^52^. The *zur* gene of J96 together with the intergenic region located upstream of the *zur* ORF was PCR-amplified using the primers Zur-*Bam*HI and Zur-*Eco*RI. The PCR-amplified fragment was cloned in pBR322 resulting the plasmid pBRzur.

### SDS-PAGE and Western immunoblotting analyses

The standard SDS-PAGE procedure was used to monitor HlyA production. Gels were routinely stained with Coomassie blue. For immunoblotting, proteins were transferred to PVDF membranes and detected with the monoclonal anti-α-hemolysin H10^53^ and a horseradish peroxidase-conjugate antibody (Promega) using the ECL Plus Western Blotting Detection System (GE Healthcare). Gels were analyzed on a Chemidoc System (BioRad) equipped with the QuantityOne® Software.

### β-Galactosidase assay

β-Galactosidase activity was monitored by the method described earlier^54^. Data shown are mean values and standard deviation of duplicate determinations from three independent experiments.

### Assay of hemolytic activity

Quantitative hemolytic assay was performed essentially as previously described^55^. Briefly, a 10% defibrinated sheep blood suspension was prepared in 0.9% NaCl containing 10 mM CaCl_2_, centrifuged and resuspended in the same volume of the same solution. The wash step was repeated three times in order to eliminate debris from broken cells. Bacterial cultures were grown in LB_10_ at 37 °C to an OD_600 nm_ of 1.0, centrifuged to discard supernatant and the bacterial pellet resuspended in the same volume of LB_10_. In 96-well microtiter plates 50 µl of blood suspension was mixed with an equal amount of a 1/125 dilution of the bacterial cell suspension, centrifuged (400 g, 10 min, 4 °C) and incubated for 60 min at 37 °C. Thereafter, 100 µl of ice-cold 0.9% NaCl was added and the microtiter plate was centrifuged (400 g, 10 min, 4 °C). A 100 µl aliquot of the supernatant was removed to another plate and the release of haemoglobin measured spectrophotometrically at 545 nm. In all plates non-infected wells were used as controls and the resulting OD_545 nm_ from those controls was subtracted to the value obtained for the different samples. A bar shows the arithmetic mean of experimental results and the error bar indicates the standard deviation from three independent experiments. Control experiments using blood suspension prepared in 0.9% NaCl, in the absence of CaCl_2_, were performed. Consistent with the fact that the activity of hemolysin is calcium-dependent no hemoglobin release was detected. LB_10_ was used since regular LB (5 g/L NaCl) causes unspecific release of haemoglobin due to osmotic pressure alterations.

### T24 cell culture assays

T24 bladder epithelial cells were maintained in RPMI supplemented with 10% heat-inactivated fetal bovine serum (FBS) and 1% penicillin-streptomycin. Bacterial cultures were grown in LB at 37 °C. Infection assays were performed in 24-well plates containing RPMI- 10% FBS where 10 µL of bacterial growth (OD_600 nm_ of 0.5) were added to a monolayer of T24 cells. After infection, the cells were incubated at 37 °C, 5% CO2 for 2 h for microscopy analysis and for 3.5 h for measurements of cell detachment. The T24 monolayers were visualized after 2.5 h with an Axiovert 40 C inverted optical microscope (Carl Zeiss) and images were captured with EOS 1000D Canon camera at 25X increase. After 3.5 h infection, the cells were washed three times with ice-cold PBS, fixed in 10% formalin for 15 min at room temperature and stained in 0.1% Crystal Violet, 0.2% ethanol for 10 min at room temperature. After 2 washes with water, the cells were allowed to dry at room temperature and then lysed in 2% sodium dodecyl sulphate (SDS) for 30 min. The absorbance was quantified by spectrophotometry at 590 nm as an indicator of crystal violet staining. The data is given as percentage of attached cells. For each experiment the OD_590 nm_ of non-infected samples was given arbitrarily the value 100%. A bar shows the arithmetic mean of experimental results and the error bar indicates the standard deviation from three biological replicates.

### Random mutagenesis with pBT20 plasmid

Mutagenesis experiments were performed using the mariner-based transposon system carried on plasmid pBT20^56^. Bacterial suspensions of the donor (*E*. *coli* S17-λpir containing pBT20) and the recipient (JFV3) were recovered from overnight plates and resuspended in LB. These suspensions were adjusted to OD_600 nm_ of 40 and 20, respectively, and mixed in a 1:1 ratio. Aliquots of 50 μl were spotted on a LB agar plate and incubated at 37 °C for 6 h. The mating mixtures were subsequently recovered in LB and cultured on LB agar plates containing kanamycin, gentamicin, and X-gal at 37 °C. Clones depicting darker blue color were selected, and *hly*_*II*_ transcriptional expression was monitored by β-galactosidase quantification. Clone #13 genomic DNA was extracted using the Blood & Cell Culture DNA Midi kit (Qiagen) with the Genome DNA Buffer Set (Qiagen) and sequenced using the primer 526.

### 5′RACE assay

The 5′ end of the *hly*_*II*_ operon transcript was determined using the 5′ RACE System for Rapid Amplification of cDNA Ends (Invitrogen). Total RNA was isolated from cultures of the J96 strain grown in LB at 37 °C up to an OD_600 nm_ of 1.0. Retrotranscription was done using the oligo hlyIIBamIII, specific for the *hly*_*II*_ operon. Next, a polyC tail was added to the 3′ end of the cDNA by using terminal deoxynucleotidyl transferase. Two rounds of PCR amplification were performed using specific primers for the *hly*_*II*_ operon, hlyII-GSP2 and hlyII-GSP3, and primers provided by the commercial kit. The final PCR product was isolated and sequenced.

### Electrophoretic Mobility Shift Assay

Zur binding to the Zur box of the J96 *hly*_*II*_ operon was assessed as previously reported^31^. A fluorescence-labeled 50-bp fragment of the *hly*_*II*_ promoter containing the putative Zur binding site was prepared by annealing two single strand DNA oligos. An oligo with a 5′ end Cy5 label (5′-/5Cy5/TCTTTATTTATTGTTTGTGTTATGTTATAACATATAAAAGAGTATTGTTT-3′) and its reversed complement sequence with no labels (5′-AAACAATACTCTTTTATATGTTATAACATAACACAAACAATAAATAAAGA-3′) were ordered from Integrated DNA Technologies (IDT). To make double stranded DNA, oligos were dissolved in the annealing buffer (10 mM Tris, 1 mM EDTA, 50 mM NaCl, pH 7.5), incubated at 95 ^o^C for 5 min and slowly cooled to room temperature. 60 pM of DNA and various concentrations of the wild-type Zur protein (from *E*. *coli* MG1655) was mixed in the binding buffer (10 mM Tris, 10 mM NaCl, pH 8.0) containing 2 mM MgCl_2_, 1 mM CaCl_2_, 166 mM KCl, 100 mM L-potassium glutamic acid, 100 µg/mL bovine serum albumin (BSA), 2 µg/mL sonicated salmon sperm DNA, 5 mM DTT, 50 µM ZnSO_4_, 5% glycerol. DNA-protein mixtures were incubated at room temperature shielded from light for 30 minutes. The purity of the Zur protein used is shown in Fig. S5. In all Zur/DNA binding assays, protein samples were equilibrated with operator DNA with addition of excess non-specific competitor DNA (i.e. salmon sperm DNA). This allows direct monitoring of specific protein-DNA interactions. Samples were then resolved in a 10% polyacrylamide (37.5:1 acrylamide:bisacrylamide) gel. Both gel and electrophoresis buffers contain 89 mM Tris (pH 8.0), 89 mM boric acid and 50 µM ZnSO_4_. Gels were run at 10 V/cm for 60–70 minutes and visualized by Typhoon 9400 imager (GE Healthcare).

### Statistical analysis

Analysis was carried out using R software. Unpaired t tests with p-values adjusted by Bonferroni’s method for multiple comparisons were used to make comparisons between two groups from a data set formed by several groups (i.e. cell detachment and cytolysis data). One-way analysis of variance (ANOVA) with Tukey’s multiple comparisons test was used for comparison of a data set formed by more than two groups (i.e. complementation assay). Two-way ANOVA was used to compare a data set with two variables (i.e. *hly*_II_ expression in several genotypes under different zinc concentrations). A value of *P* < 0.05 was considered statistically significant.

### Data availability

All data generated or analyzed during this study are included in this published article (and its Supplementary Information files).

## Electronic supplementary material

Supplementary information
