# Supplementary material for: A new role for Zinc limitation in bacterial pathogenicity: modulation of α-hemolysin from uropathogenic Escherichia coli
Source: Sci Rep. 2018 Apr 25;8:6535. doi: 10.1038/s41598-018-24964-1 (PMC5916954; doi:10.1038/s41598-018-24964-1)
Supplement: Supplementary file 1 — Supplementary information [file 41598_2018_24964_MOESM1_ESM.pdf]

**A new role for Zinc limitation in bacterial pathogenicity: modulation of α-hemolysin from uropathogenic *Escherichia coli*.**

Elsa Velasco, Suning Wang, Marianna Sanet, Jorge Fernández-Vázquez, Daniel Jové, Estibaliz Glaría, Annabel F. Valledor, Thomas V. O'Halloran and Carlos Balsalobre.

**Supplementary information**

Table S1. Primers used in this work 2

Figure S1. Electrophoretic analysis of secreted protein extracts of J96, JFV16 (J96ΔII) and JFV21 (J96ΔI) 3

Figure S2. *zur* mutation causes derepression of the $hly_{II}$ operon expression in cultures grown up to mid-log phase 4

Figure S3. Alignment of the $hly_{II}$ Zur binding site to the Zur consensus sequence 5

Figure S4. Effect of Δ*zur* mutation in the expression of α-hemolysin in the UPEC strain CFT073 6

Figure S5. Pure Zur protein 7

Figure S6. Original gel image used to create Fig. 1 c panel 8

Figure S7. Original gel and blot images used to create Fig. 2 c panel 9

Figure S8. Original gel images used to create Fig. 4 c panel 10

Figure S9. Original gel image used to create Fig. 6d panel 11

**Table S1. Primers used in this study.**

| | |
|---|---|
| HlyA-P1 | 5'- ATCATATCCATTTTCAAAGTAATTTTTGCCGTGTTTTGTG TAGGCTGGAGCTGCTTC -3' |
| HlyA-P2 | 5'- CAAATTAAAAGCACACACTGCAGTCTGCAAAGCAATCCG CATATGAATATCCTCCTTAGT -3' |
| Hly1-P1 | 5'- CCTGATTAATTCAACCCCTTATCTGACAGGGGTAGAAGT GTAGGCTGGAGCTGCTTC -3' |
| Hly1-P2-2 | 5'- ATTCAGCAATAACATCGGCCATTTTACTGGCGACATGCA TATGAATATCCTCCTTAGT -3' |
| Zur-P1 | 5'- CCATGTGCTTCAATAACATTATGCCGCAGGGCAAAACCC ATTGTGTAGGCTGGAGCTGCTTCG -3' |
| Zur-P2 | 5'- CACCCTGTTCTCGGCGTTTAAGTGAGAACTATGGTAAAG TCATATGAATATCCTCCTTA -3' |
| Zur-P1.2 | 5' – TTACCGCTGGTAATTAATCCCTCCTGCCCGACATGTAC AAGGGTGTAGGCTGGAGCTTCG -3' |
| Zur-P2.1 | 5'- GCTACTATAGCATAACGAATATGCGATCAGGCAATGTGAC CATATGAATATCCTCCTTAGT -3' |
| 526 | 5'- CTGTATGGAACGGGATG -3' |
| HlyII-BamIII | 5'- GGATCCATGCCCAAGAACCTC -3' |
| HlyII-GSP2 | 5'- TAAAAAGATCACATAAAGAAATGG -3' |
| HlyII-GSP3 | 5'- CCTGCACCTTGCTTCATTTG -3' |
| Zur-*Bam*HI | 5'- GGATCCCATCGGGGCAATCGAGAAAC -3' |
| Zur-*Eco*RI | 5'- GAATTCCAGGAACGCTACGGTTATC -3' |

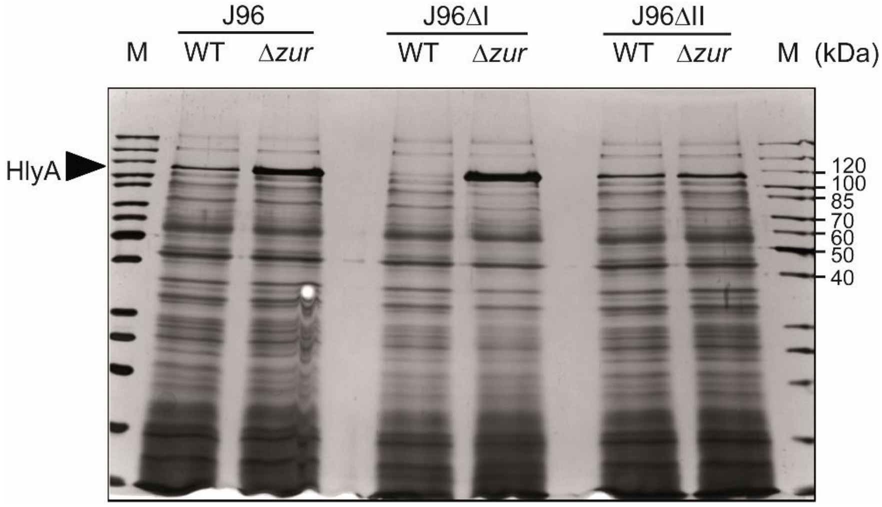


**Figure S1**. **Electrophoretic analysis of secreted protein extracts of J96, JFV16 (J96ΔII) and JFV21 (J96ΔI)**. Bacterial cultures were grown in LB at 37 ºC up to late-log phase ($OD_{600nm}$ of 1.0). Secreted extracts were analyzed in silver stained 10 % SDS-PAGE. The band corresponding to HlyA is indicated with an arrowhead. Lanes M: molecular mass markers (size indicated in kDa).

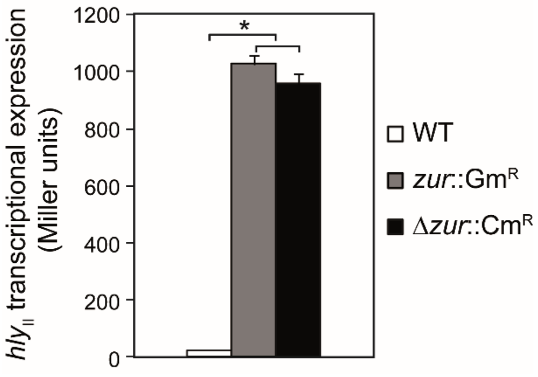


**Figure S2. *zur* mutation causes derepression of the *hly*$_{II}$ operon expression in cultures grown up to mid-log phase.** Transcriptional expression of the *hly*$_{II}$ operon from cultures of the JFV3 strain (J96 *hlyII::lacZ*) and its *zur*::Gm$^R$ and *zur*::Cm$^R$ derivatives. Cultures were grown in LB at 37ºC up to mid-log phase ($OD_{600nm}$ of 0.4). β-galactosidase activity (Miller units) was determined in three independent cultures. Mean values with standard deviations are plotted. * $P < 0.05$, ANOVA with Tukey's multiple comparisons test.

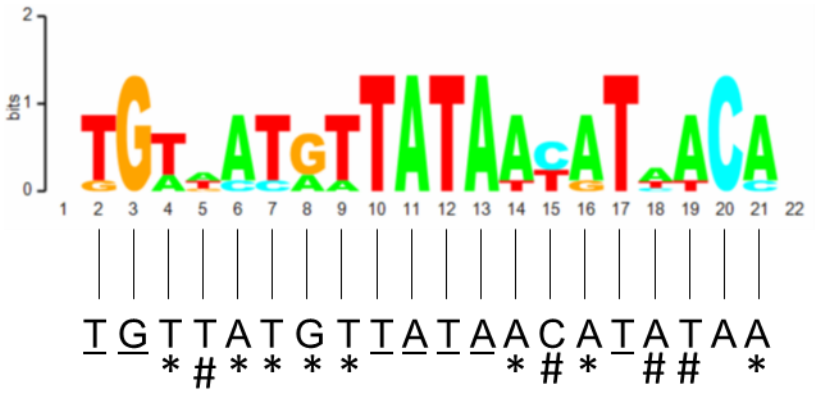


**Figure S3. Alignment of the *hly*$_{II}$ Zur binding site to the Zur consensus sequence[1].** The *hly*$_{II}$ Zur binding site was aligned with the sequence logo representation of the template strain in Zur-DNA recognition based on the following *E. coli* Zur operators: *znuA*, *znuC*, *zinT*, and *l31p*. Underlined bases indicate identical to the consensus residues, bases with * are identical to the most predominant residue and bases labelled with # are identical with no predominant residues.

1 Gilston, B. A. *et al.* Structural and Mechanistic Basis of Zinc Regulation Across the E. coli Zur Regulon. *PLoS Biol.* **12,** e1001987 (2014).

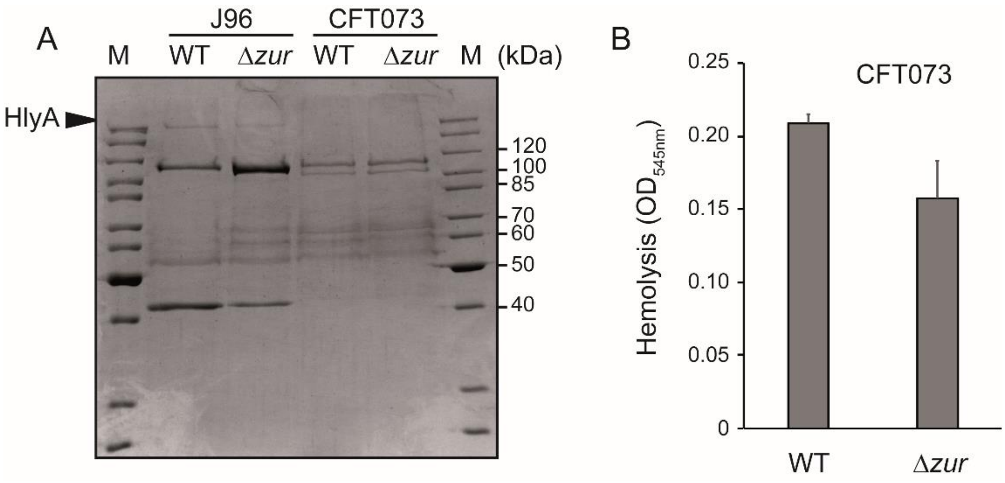


**Figure S4. Effect of Δ*zur* mutation in the expression of α-hemolysin in the UPEC strain CFT073.** A. Electrophoretic analysis of secreted protein extracts of J96, EV27 (J96Δ*zur*), CFT073 and DJ1 (CFT073Δ*zur*). Bacterial cultures were grown in LB at 37 ºC up to late-log phase ($OD_{600nm}$ of 1.0). Secreted extracts were analyzed in Coomassie blue stained 10 % SDS-PAGE. The band corresponding to HlyA is indicated with an arrowhead. Lanes M: molecular mass markers (size indicated in kDa). B. Hemolysis after 1 hour infection of sheep blood cell suspensions with CFT073 and DJ1 (CFT073Δ*zur*). The release of haemoglobin was monitored by $OD_{545nm}$.

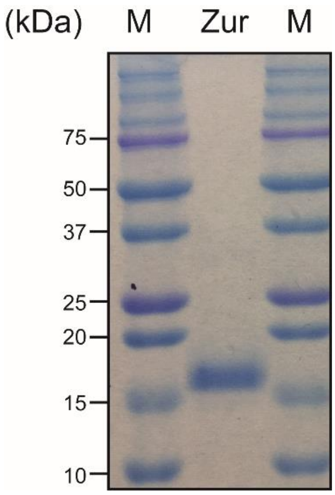


**Figure S5. Pure Zur protein.** 5 μg of pure Zur protein was analysed in 12% SDS-PAGE, stained by SimpleBlue safe stain (Invitrogen). Lanes M: molecular mass markers (size indicated in kDa).

Coomassie stained gel

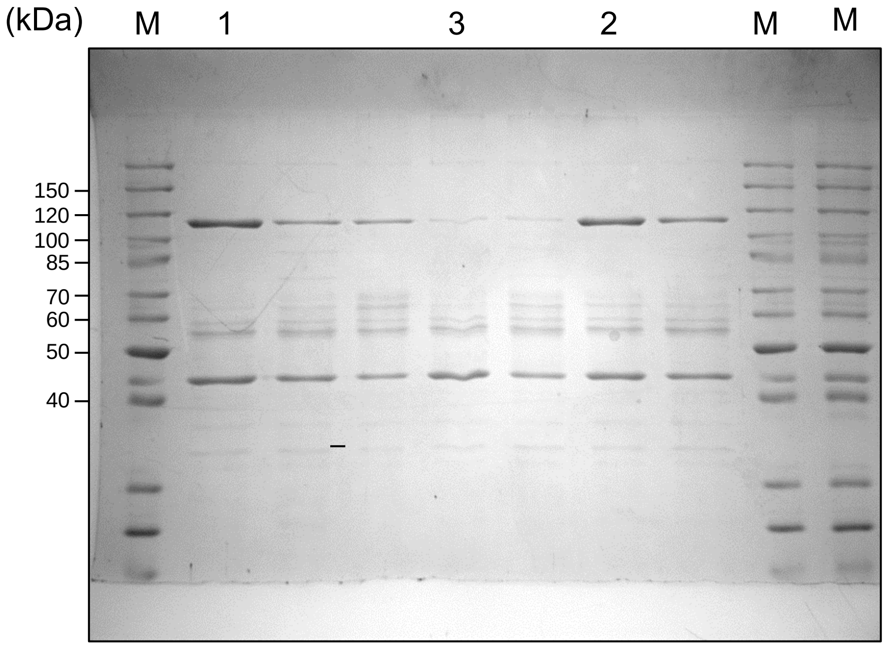


Lane 1: Cell-free extract from J96
Lane 2: Cell-free extract from J96ΔII
Lane 3: Cell-free extract from J96ΔI
Lanes M: molecular mass markers (size indicated in kDa).

**Figure S6. Original gel image used to create Fig. 1c panel.**

Coomassie stained gel

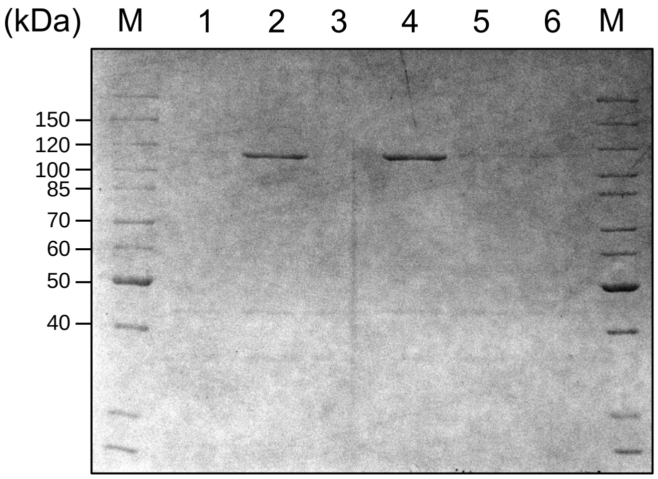


Lane 1: Cell-free extract from J96
Lane 2: Cell-free extract from J96ΔzurLane 3: Cell-free extract from J96ΔI
Lane 4: Cell-free extract from J96ΔIΔzur
Lane 5: Cell-free extract from J96ΔII
Lane 6: Cell-free extract from J96ΔIIΔzur
Lanes M: molecular mass markers (size indicated in kDa).

Western blots anti-HlyA

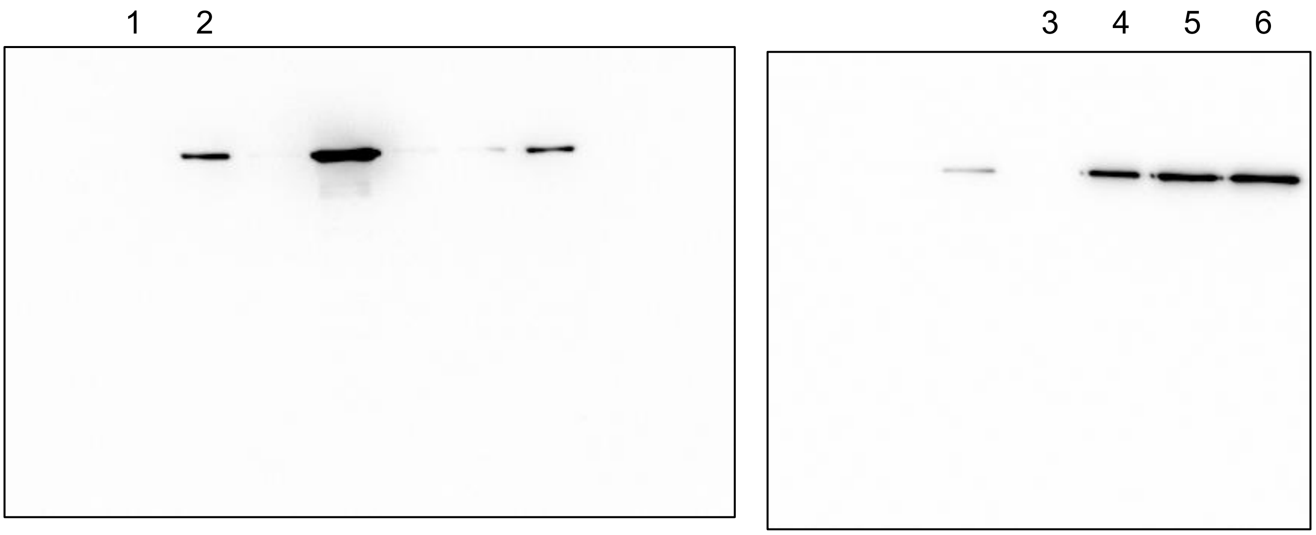


Lane 1: Cell-free extract from J96
Lane 2: Cell-free extract from J96Δzur
Lane 3: Cell-free extract from J96ΔI
Lane 4: Cell-free extract from J96ΔIΔzur
Lane 5: Cell-free extract from J96ΔII
Lane 6: Cell-free extract from J96ΔIIΔzur

**Figure S7. Original gel and blot images used to create Fig. 2c panel.**

Coomassie stained gels

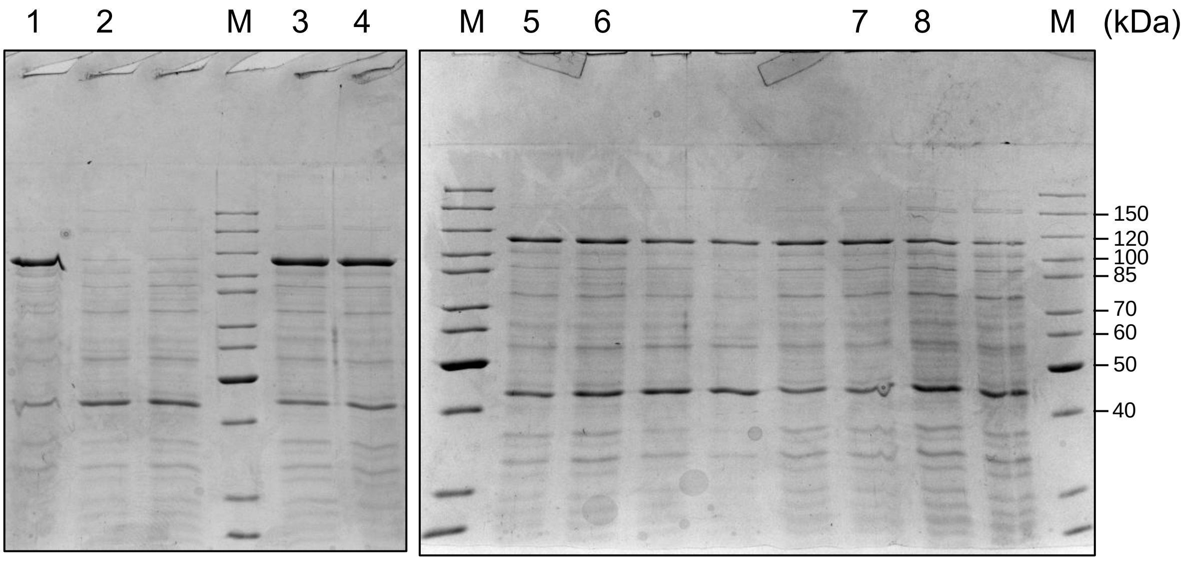


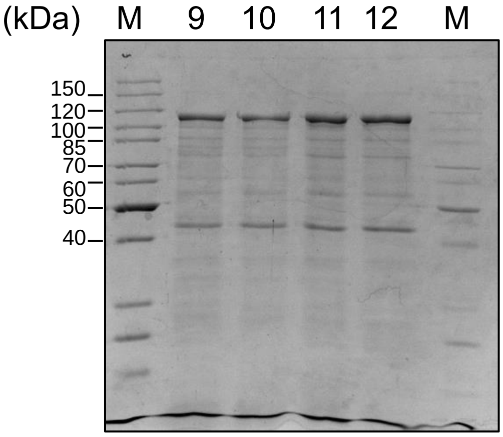


Lane 1: Cell-free extract from J96ΔI (- $ZnCl_2$)
Lane 2: Cell-free extract from J96ΔI (+ $ZnCl_2$)
Lane 3: Cell-free extract from J96ΔIΔzur (- $ZnCl_2$)
Lane 4: Cell-free extract from J96ΔIΔzur (+ $ZnCl_2$)
Lane 5: Cell-free extract from J96ΔII (- $ZnCl_2$)
Lane 6: Cell-free extract from J96ΔII (+ $ZnCl_2$)
Lane 7: Cell-free extract from J96ΔIIΔzur (- $ZnCl_2$)
Lane 8: Cell-free extract from J96ΔIIΔzur (+ $ZnCl_2$)
Lane 9: Cell-free extract from J96 (- $ZnCl_2$)
Lane 10: Cell-free extract from J96 (+ $ZnCl_2$)
Lane 11: Cell-free extract from J96Δzur (- $ZnCl_2$)
Lane 12: Cell-free extract from J96Δzur (+ $ZnCl_2$)
Lanes M: molecular mass markers (size indicated in kDa).

**Figure S8. Original gel images used to create Fig. 4c panel.**

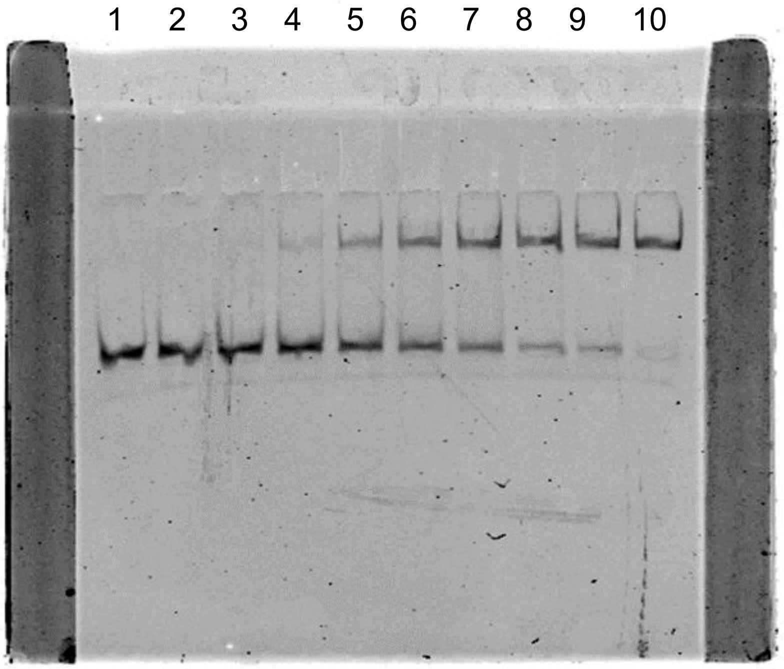


Lane1: absence of Zur

Lane 2: 0.015 nM Zur dimer

Lane 3: 0.03 nM Zur dimer

Lane 4: 0.05 nM Zur dimer

Lane 5: 0.1 nM Zur dimer

Lane 6: 0.15 nM Zur dimer

Lane 7: 0.2 nM Zur dimer

Lane 8: 0.3 nM Zur dimer

Lane 9: 0.35 nM Zur dimer

Lane 10: 0.9 nM Zur dimer

**Figure S9. Original gel image used to create Fig. 6d panel.**
